# Outcomes of endoscopic transcapsular iliopsoas tenotomy for snapping hip syndrome: Minimum 10‐year follow‐up

**DOI:** 10.1002/jeo2.70543

**Published:** 2026-02-09

**Authors:** Mattia Loppini, Marco Minelli, Katia Chiappetta, Francesco La Camera, Guido Grappiolo, Federico Della Rocca

**Affiliations:** ^1^ Department of Biomedical Sciences Humanitas University Milan Italy; ^2^ IRCCS Humanitas Research Hospital Milan Italy; ^3^ Fondazione Livio Sciutto Onlus Campus Savona ‐ Università degli Studi di Genova Savona Italy

**Keywords:** arthroscopy, hip, iliopsoas, long‐term, tenotomy

## Abstract

**Purpose:**

This study aims to evaluate the clinical outcomes of patients who underwent endoscopic transcapsular iliopsoas tenotomy for painful snapping hip syndrome at minimum of ten years follow‐up.

**Methods:**

This is a monocentric retrospective study on a consecutive series of patients undergoing endoscopic transcapsular iliopsoas tenotomy procedure for painful snapping hip syndrome between January 2012 and June 2015. Included patients were clinically evaluated at a minimum of ten years of follow‐up. Perioperative, early or late complications and adverse events were recorded. Modified Harris Hip Score (mHHS) and Hip Disability and Osteoarthritis Outcome Score (HOOS) were calculated preoperatively, at 1 year, and after 10 years postoperatively. Hip flexion strength was assessed preoperatively and at 1 month, 6 months, 1 year and after 10 years postoperatively.

**Results:**

Twenty patients were included in the study. No patient was lost to follow‐up at a minimum of 10 years. Mean follow‐up was 10.6 years. No perioperative complications were reported. No serious or minor adverse events were recorded. None of the patients required revision hip arthroscopy or was scheduled for total hip arthroplasty at the last follow‐up. Three patients (15%) exhibited hip flexion weakness on clinical examination and in these patients MRI revealed iliopsoas muscle atrophy. Three patients (15%) reported persistent snapping during hip flexion‐extension. No sensory deficits were observed. Both mHHS and HOOS were significantly improved (*p* < 0.001) at the last follow‐up.

**Conclusions:**

Endoscopic transcapsular iliopsoas tenotomy is a safe and effective procedure for snapping hip syndrome at long‐term follow‐up.

**Level of Evidence:**

Level IV.

**Abbreviations**
BMI: body mass index
HOOS: Hip Disability and Osteoarthritis Outcome Score
LCEA: lateral centre‐edge angle
mHHS: modified Harris Hip Score
MRI: magnetic resonance imaging

## INTRODUCTION

The iliopsoas is a complex musculotendinous unit formed by three different muscles: the psoas major, the psoas minor and the iliacus [4]. The iliopsoas plays a role in a number of physiological functions as hip flexion, femur external rotation, lateral and anterior flexion and stabilisation of the trunk [4]. Disorders related to this musculotendinous unit represent a common cause of anterior hip pain, to the point that they are responsible for 12%–36% cases of chronic groin pain among athletes [30]. Among these conditions, a common disorder is represented by ‘snapping hip syndrome’ or ‘coxa saltans’, which has been described clinically as the painful snapping sensation resulting from iliopsoas tendon impingement over deeper structures during hip extension [9, 31]. This condition is commonly associated to anterior acetabular labrum tears secondary to traction or compression by the iliopsoas tendon [9, 31]. The first approach for snapping hip syndrome is conservative, based on a tendon stretching routine and local corticosteroid injections [9, 31]. Whenever conservative approaches fail to relieve symptoms, surgical treatment could be indicated: both open and arthroscopic techniques have been described [28]. However, open surgery techniques recorded a 40% rate of complications at mid‐term follow‐up, among which recurrent snapping, sensory deficit and persistent hip flexion weakness were reported [17]. Therefore, endoscopic techniques were developed to release the iliopsoas tendon [14, 16, 21, 23]. The endoscopic iliopsoas tendon release can be performed inside the joint (transcapsular) at the level of the acetabular rim (central compartment), or outside the joint capsule (extracapsular) targeting the tendon at its insertion on the lesser trochanter (peripheral compartment) [20]. Transcapsular tenotomy was associated with a lower rate of hip flexor weakness when compared to release at the lesser trochanter, likely because it is performed within the central compartment thus the muscular portion of the tendon remains preserved [20]. Although, this approach carries a potentially increased risk of femoral nerve branches injuries, since they overlie the iliopsoas at this level [20]. However, long‐term outcomes of the endoscopic tenotomy of the iliopsoas have not been described yet. Therefore, the aim of the current study was to evaluate the long‐term outcomes of transcapsular tenotomy of the iliopsoas tendon. The hypothesis of this study is that endoscopic transcapsular iliopsoas tenotomy would result in significant and durable improvement in patient‐reported functional outcomes with a low rate of complications at long‐term follow‐up. The primary outcome of interest was the change in clinical outcome scores over time, specifically the modified Harris Hip Score (mHHS) and Hip Disability and Osteoarthritis Outcome Score (HOOS), from baseline to final follow‐up.

## MATERIALS AND METHODS

This is a monocentric retrospective study on a consecutive series of patients undergoing transcapsular iliopsoas partial tenotomy procedure during hip arthroscopy between January 2012 and June 2015. These patients underwent transcapsular iliopsoas partial tenotomy for a clinically evident painful snapping hip syndrome resistant to conservative therapies for at least six months, with or without concomitant clinical and radiological findings of intra‐articular pathologies. Patients who previously underwent hip arthroscopy or total hip arthroplasty on the same hip were excluded from the study. Included patients were clinically evaluated at a minimum of ten years of follow‐up. Appropriate ethical approval was achieved from the Institutional Board Committee (protocol number 618/17 IRCCS Istituto Clinico Humanitas). Informed consent was obtained from all the participants included in the study.

### Surgical procedure

**Figure 1:**
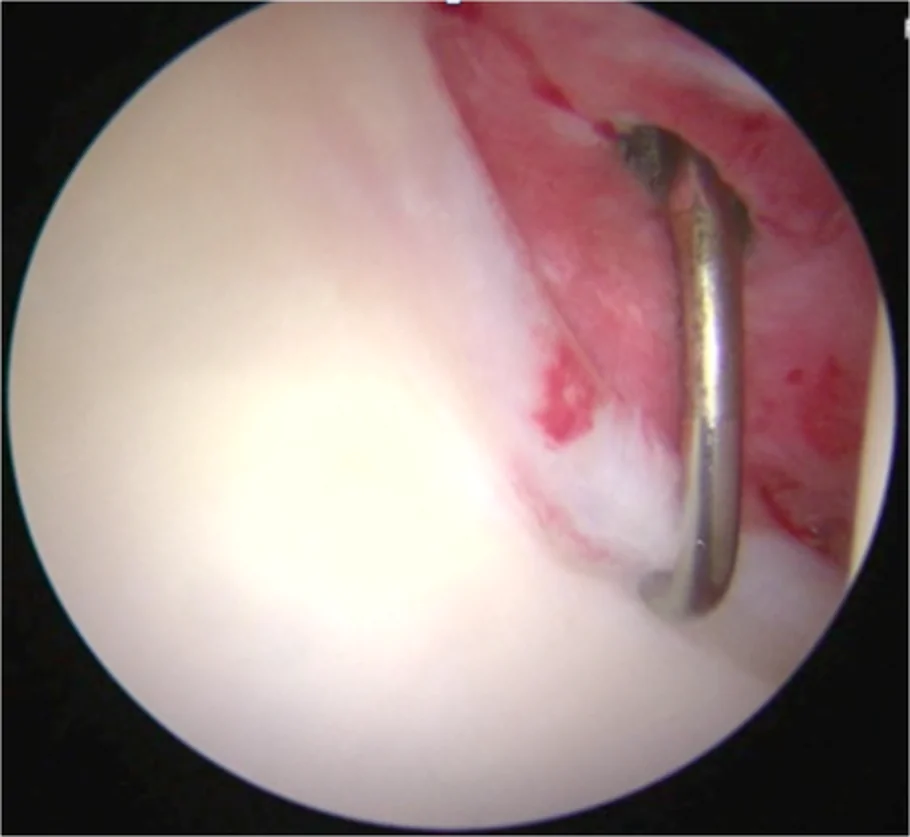
Arthroscopic view of the anterior capsulo‐labral complex at the 3 o'clock position, demonstrating degeneration and inflammation of the capsulo‐labral complex.

**Figure 2:**
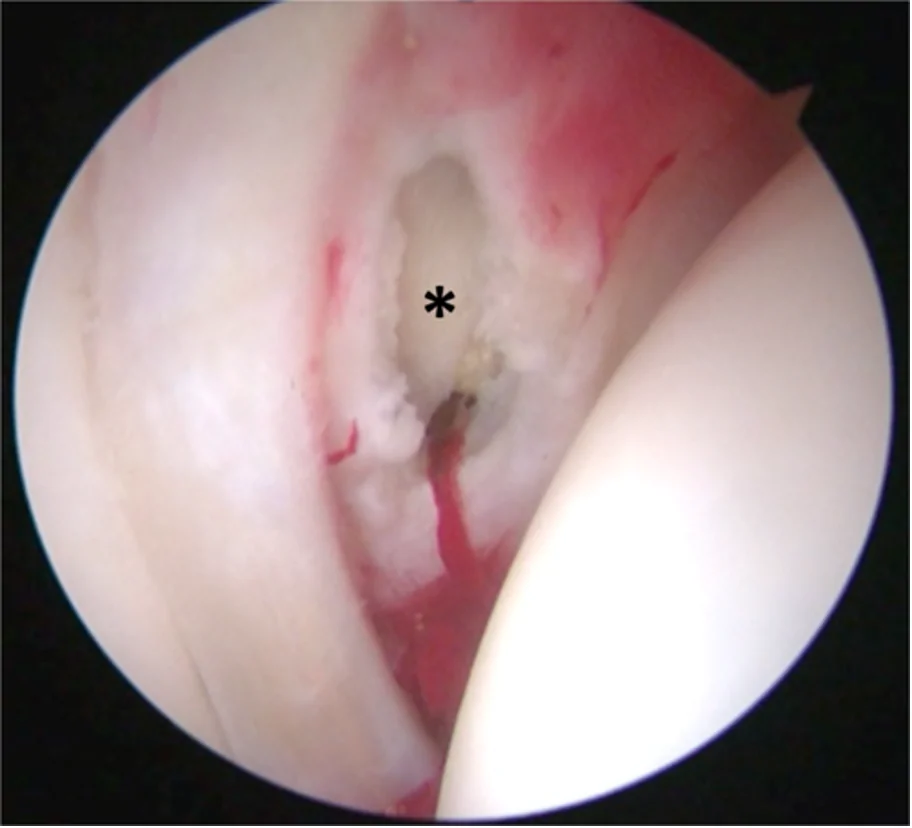
Arthroscopic view of an anteromedial capsulotomy performed at the 3 o'clock position, at the level of the psoas valley, allowing visualisation of the iliopsoas tendon fibres (*) located posterior to the capsule.

Preoperatively, a standard anteroposterior pelvis and a modified Dunn [26] view hip radiographs, and an MRI arthrogram were requested for every patient as diagnostic work‐up for intra‐articular pathologies. Lateral centre‐edge angle (LCEA) was calculated on anteroposterior pelvis radiographs with OsiriX DICOM Viewer (Pixmeo, Switzerland) [13]. All the surgical procedures were performed by a single surgeon specialised in hip arthroscopy. Spinal loco‐regional anaesthesia was administered prior to the procedure. After positioning the patient in supine position on a traction table, a diagnostic hip arthroscopy was performed with a padded perineal post to avoid pudendal nerve and genital injuries. Access to the hip joint was obtained through the anterolateral, distal anterolateral and mid‐anterior arthroscopic portals; a 70° arthroscope was used. Diagnostic arthroscopy was performed: whenever present, intra‐articular lesions were addressed (Figure 1). Then, a medial capsulotomy was performed to expose the central compartment and visualise the iliopsoas tendon over the anterior acetabular rim and, once identified the tendon crossing the anterior capsule, a transverse incision was made through the capsule and tendon fibres via a radiofrequency device (Figure 2). After the release, dynamic manoeuvres in flexion and extension were performed under direct endoscopic visualisation to check for residual snapping. The capsule was then closed with non‐absorbable sutures. All the included patients followed the same post‐operative rehabilitation protocol. Standing was allowed on the first postoperative day. Partial weight‐bearing and passive motion machine were allowed for a month. Patients were recommended to avoid forceful hip flexion against resistance for a month to allow healing of the tenotomy site.

### Outcomes analysis

Any perioperative, early or late complications and adverse events were reported. A serious adverse event was defined as an adverse event leading to a death, injury, or permanent impairment to a body structure or a body function. A minor adverse event was defined as the presence of tenderness, redness, and oedema of the index hip at the first follow‐up visit. Clinical assessment was performed pre‐ and postoperatively by an orthopaedic specialist trained in hip surgery that was not involved in the surgical procedure. The modified Harris Hip Score (mHHS) and the Hip Disability and Osteoarthritis Outcome Score (HOOS) were employed to investigate the clinical outcomes of interest preoperatively, at 1 year, and after 10 years postoperatively [8, 29]. Patient satisfaction was documented by asking patients whether they were ‘very satisfied’, ‘satisfied’, ‘neutral’, ‘dissatisfied’ and ‘very dissatisfied’ about the procedure at the latest follow‐up. Hip flexion strength was assessed preoperatively and at 1 month, 6 months, 1 year, and after 10 years postoperatively using a clinical scale ranging from 0 to 5 (0 – no contraction, 1 – flicker or trace contraction, 2 – active movement with gravity eliminated, 3 – active movement against gravity, 4 – active movement against gravity + some resistance, 5 – normal strength against full resistance) [24]. Whenever a reduction in hip flexion strength was observed, patients underwent MRI examination to check for iliopsoas fatty infiltration from 0 to 4 based on estimated percentage of fat content in quartiles (0: 0%–5%, 1: 6%–25%, 2: 26%–50%, 3: 51%–75%, 4: >75%) according to the Quartile classification, which has been shown to demonstrate high reproducibility when applied to hip musculature [11].

### Statistical analysis

Numerical outcomes were described as mean and standard deviation or range (minimum–maximum). Discrete outcomes were described as absolute and relative frequencies. Complications and treatment failures were described as crude event proportions. The d'Agostino‐Pearson test was used to analyse the distribution of the data collected, after which a paired *t*‐test or the Mann–Whitney test was used to evaluate for statistical significance. Qualitative data were compared using the Chi‐squared test and Fisher exact test. The difference was considered statistically significant when the *p* value was <0.05. A post‐hoc power analysis for paired samples (two‐tailed, α = 0.05) was performed using the observed preoperative and postoperative means and standard deviations. Effect size for paired data (Cohen's dz) was calculated as the mean pre–post difference divided by the standard deviation of the differences (SD_diff), where SD_diff = √(SD_pre² + SD_post² − 2·r·SD_pre·SD_post). Because the pre–post correlation (*r*) was not recorded, we report the primary estimate assuming r = 0.50 and provide a sensitivity analysis for *r* = 0.30 and *r* = 0.70. Achieved power (1−β) was computed for the paired t‐test using the noncentrality parameter (dz·√n). The software IBM SPSS Statistics 21.0.0.1 (IBM Corp, Armonk, NY, USA) was used for the statistical analysis.

## RESULTS

### Patient characteristics

Twenty patients met the inclusion criteria and were finally included in the study. No patient was lost to minimum 10‐year follow‐up. The follow‐up was available for all patients with a mean value of 10.6 years (range 10.0–13.0 years). The final cohort was composed by 17 females and 3 males, with an average age of 42.4 years old (range 24.0–55.0 years). Mean BMI was 24.0 (range 19.0–34.4). Mean LCEA was 38.9° (range 29.1°–54.1°). Mean surgical time was 93.5 minutes (range 43.0–196.0 minutes). The procedure was performed on the right hip in eight patients (40%) and on the left hip in twelve patients (60%). Eighteen patients (90%) underwent concomitant intra‐articular procedures, which are listed in Table 1.

**Table 1:** Patients characteristics.

No.	Gender	Age	BMI	Pre‐op flexion strength	Pre‐op LCEA angle	Surgical time (min)	Surgical procedure
1	F	37.0	27.3	5	35.2°	43.0	Iliopsoas release
2	M	44.0	24.0	5	41.2°	67.0	Iliopsoas release + anterosuperior labral resection
3	M	39.0	25.1	5	35.4°	112.0	Iliopsoas release + anterosuperior labral repair + femur neck reshaping
4	F	50.0	24.1	5	40.1°	145.0	Iliopsoas release + anterosuperior labral repair + femur neck reshaping
5	F	40.0	24.8	5	36.7°	73.0	Iliopsoas release + femur neck reshaping
6	F	43.0	24.8	5	33.3°	100.0	Iliopsoas release + femur neck reshaping
7	F	41.0	34.4	5	48.1°	127.0	Iliopsoas release + acetabular trimming
8	F	47.0	23.95	5	54.1°	95.0	Iliopsoas release + acetabular trimming + femur neck reshaping
9	F	46.0	22.27	5	34.3°	93.0	Iliopsoas release + anterosuperior labral repair + femur neck reshaping
10	F	24.0	26.35	4	29.1°	49.0	Iliopsoas release
11	F	38.0	23.12	5	38.3°	101.0	Iliopsoas release + femur neck reshaping
12	F	55.0	25.39	5	43.3°	60.0	Iliopsoas release + femur neck reshaping
13	F	32.0	19.05	5	43.1°	75.0	Iliopsoas release + femur neck reshaping
14	M	37.0	19.01	4	33.7°	113.0	Iliopsoas release + femur neck reshaping + acetabular subchondral microfractures
15	F	35.0	24.22	5	47.2°	113.0	Iliopsoas release + femur neck reshaping + acetabular trimming
16	F	55.0	27.34	5	37.0°	196.0	Iliopsoas release + femur neck reshaping
17	F	43.0	23.31	5	45.2°	66.0	Iliopsoas release + femur neck reshaping
18	F	51.0	21.97	5	31.1°	53.0	Iliopsoas release + femur neck reshaping
19	F	43.0	19.71	5	35.1°	115.0	Iliopsoas release + anterosuperior labral repair
20	F	49.0	20.7	5	36.6°	74.0	Iliopsoas release + anterosuperior labral repair + femur neck reshaping

Abbreviations: BMI, body mass index; LCEA, lateral centre‐edge angle.

### Clinical outcomes

No perioperative complications were reported. No serious or minor adverse events were recorded. None of the patients required revision hip arthroscopy or was scheduled for total hip arthroplasty at the last follow‐up. At 1 month postoperatively, eight patients (40%) demonstrated hip flexion weakness: one decreased from grade 5 to 3 (active movement against gravity), five from grade 5 to 4 (against gravity with some resistance), and two remained at grade 4, unchanged from baseline. By 6 months, the two patients with persistent preoperative grade 4 strength and three others recovered to grade 5 (normal strength against full resistance). At the 1‐year follow‐up, strength outcomes were stable compared with the 6‐months evaluation, with no additional cases of weakness or recovery. At the latest follow‐up, persistent hip flexion weakness was observed in three patients (15%): one graded 3 and two graded 4 (Table 2). In these patients, MRI revealed reduction in iliopsoas muscle volume: fatty infiltration was graded in one patient as 3 – severe infiltration (51%–75%), and in the other ones as 2 – moderate infiltration (26‐50%). Three patients (15%) reported persistent snapping during hip flexion‐extension. None of the patients reported sensitive defects.

**Table 2:** Hip flexion strength outcomes.

No.	Pre‐op flexion strength	1 month post‐op flexion strength	6 months post‐op flexion strength	1 year post‐op flexion strength	>10 years post‐op flexion strength
1	5	5	5	5	5
2	5	5	5	5	5
3	5	3	3	3	3
4	5	5	5	5	5
5	5	5	5	5	5
6	5	5	5	5	5
7	5	5	5	5	5
8	5	5	5	5	5
9	5	5	5	5	5
10	4	4	5	5	5
11	5	4	4	4	4
12	5	5	5	5	5
13	5	5	5	5	5
14	4	4	5	5	5
15	5	5	5	5	5
16	5	5	5	5	5
17	5	4	4	4	4
18	5	5	5	5	5
19	5	5	5	5	5
20	5	5	5	5	5

All the analysed scores were significantly improved (*p* < 0.001) at the last follow‐up. Modified Harris Hip score increased from a pre‐operative value of 63.7 ± 5.9 to 86.1 ± 7.9 at the 1‐year follow‐up, and to 88.3 ± 6.2 at the last follow‐up. Similarly, HOOS improved from a baseline value of 71.5 ± 7.2 to 85.9 ± 6.4 at the 1‐year follow‐up, and to 86.7 ± 5.9 at the last available follow‐up. For mHHS (mean change 24.6; SD_pre 5.9; SD_post 6.2; *n* = 20), assuming *r* = 0.50 gave SD_diff = 6.06 and Cohen's dz = 4.06, yielding power >0.99. Sensitivity across plausible r values showed dz = 3.43 (*r* = 0.30; SD_diff = 7.16; power >0.99) to dz = 5.24 (*r* = 0.70; SD_diff = 4.69; power >0.99). For HOOS (mean change 15.2; SD_pre 7.2; SD_post 5.9; *n* = 20), assuming *r* = 0.50 gave SD_diff = 6.65 and Cohen's dz = 2.29, yielding power >0.99. Sensitivity showed dz = 1.94 (*r* = 0.30; SD_diff = 7.82; power >0.99) to dz = 2.92 (*r* = 0.70; SD_diff = 5.21; power >0.99). Regarding patient satisfaction, eight patients (40%) reported being ‘very satisfied’ with the surgical outcome, eleven (55%) were ‘satisfied,’ and one patient (5%) expressed to be ‘neutral’.

## DISCUSSION

This study demonstrates that endoscopic transcapsular iliopsoas tenotomy provides significant and sustained improvement in clinical outcomes over a long‐term follow‐up period of over ten years. Both the modified Harris Hip Score (mHHS) and the Hip Disability and Osteoarthritis Outcome Score (HOOS) showed statistically significant increases, reflecting functional recovery and pain relief sustained over time. The large pre–post improvements in both mHHS and HOOS produced achieved power >0.99 under a range of plausible within‐subject correlations, supporting the robustness of these findings.

Given the elective nature of the procedure and the often young and active patient population, patients satisfaction was investigated. The subjective satisfaction rates further reinforce this procedure effectiveness, with 95% of patients reporting high satisfaction or satisfaction. The reported satisfaction rates also suggest that the clinical improvements observed were meaningful to patients in their daily lives, not just statistically significant.

These findings align with previously reported short‐ to mid‐term outcomes for iliopsoas tenotomy [4, 9, 14, 19, 23], but the present study contributes valuable data by extending follow‐up beyond a decade, offering a clearer perspective on the durability of surgical benefits. In particular, all the studies reported significant improvement in outcome scores and no severe postoperative complications were reported. Accordingly, also in this study endoscopic transcapsular iliopsoas tenotomy appeared to be a safe procedure, associated with a low complication rate: no serious perioperative or postoperative adverse events were recorded. Moreover, no patient required revision hip arthroscopy or progressed to total hip arthroplasty. This is particularly relevant in younger, active populations commonly affected by iliopsoas‐related hip pathology.

However, key considerations following iliopsoas tenotomy include potential flexor weakness and radiologic atrophy, persisting snapping and iatrogenic instability. In a systematic review of the literature by Gouveia et al. [14], hip flexion weakness was observed to be present in about 49% of patients at mean follow‐up of 32 months, even though clinical outcomes and function were not significantly affected. Brandenburg et al. [4] reported an average 19% reduction in seated hip flexion strength after arthroscopic tenotomy at the labral level that was still measurable at mid‑term follow‐up, even though supine strength remained unaffected. In this series, three patients (15%) developed hip flexion weakness, corroborated by MRI findings of iliopsoas muscle atrophy. While this rate is lower than that typically associated with extracapsular release at the lesser trochanter [6, 19, 20], it underscores the importance of preserving tendon function during transcapsular release. These findings are consistent with literature suggesting that central compartment tenotomy, by maintaining more of the muscular component, may offer a more favourable strength profile compared to distal releases [6, 19, 20]. However, a limitation of this study is that only patients who reported hip flexion weakness underwent MRI evaluation. Indeed, MRI studies show atrophy of the iliopsoas muscle in up to 85%–92% of cases after iliopsoas release, both at the central and peripheral compartment [15, 32]. Even if no direct correlation was detected between MRI‐detected atrophy and clinical weakness or pain in the majority of patients, this could be clinically significant in high‐demand patients. Notably, at 1 month postoperatively, 40% of patients exhibited hip flexion weakness, but most recovered within the first 6 months, including the two cases with persistent preoperative grade 4 strength. Thus, at the latest follow‐up, only three patients (15%) demonstrated residual weakness. This pattern suggests that early postoperative weakness was largely transient, with persistent deficits limited to a small subset of patients. It is possible that these early findings were influenced by pain inhibition rather than irreversible loss of muscle function. This mechanism whereby nociceptive input limits limits voluntary muscle activation was previously reported in iliopsoas‐related hip pathology [9, 30].

Persistent snapping was reported by 15% of patients in this study, albeit without functional limitations or patient dissatisfaction. Ilizaliturri et al. [19] observed that only one out of fourteen patients experienced recurrent snapping after central compartment iliopsoas endoscopic release. Nelson and Keane [27] recorded that three out of five patients reported persistent painful snapping after labral‐level iliopsoas release. These residual symptoms may reflect incomplete resolution of dynamic tendon impingement or other coexisting biomechanical factors not addressed by the procedure. Indeed, bifid/trifid iliopsoas tendons are found in up to 95% of individuals with snapping hip and if only one head is released, the remaining heads may still snap [3, 18, 22]. Moreover, the level of the tenotomy could influence recurrence rates: central compartment releases have higher recurrence rates due to incomplete release [7]. Plus, fibrous scar formation following iliopsoas tendon release due to the healing of the tendon stump to adjacent soft tissues could lead to recurrent snapping [5].

Finally, excess femoral antetorsion correlates with increased iliopsoas tendon excursion, thus excessive anterior movement of the tendon may contribute to symptomatic snapping in anteverted femurs [2, 12]. Indeed, patients with increased femoral antetorsion (>25°) were observed to be at greater risk for inferior clinical outcomes after arthroscopic lengthening of a symptomatic snapping psoas tendon: being an important passive and dynamic stabiliser of the hip in these patients, its release may result in a greater alteration of kinematics with high‐demand activities, particularly terminal extension and external rotation when the tendon is typically at its highest tension [12]. Thus, iliopsoas release can be associated with hip instability: surgical indications should be driven based on the balance between the clinical benefit related to the procedure and the post‐arthroscopic risk of instability [25]. In this scenario, Harris stated that in order to achieve excellent outcomes with iliopsoas fractional lengthening, surgeons must ensure capsular plication and labrum preservation, release all iliopsoas tendon bands including bifid or trifid variants, and confirm that femoral version is not excessively increased, dysplasia is not more than mild, and there is no significant soft tissue hypermobility [16]. None of the patients included in this study had an acetabular coverage (LCEA) < 25°, which is commonly recognised as the cutoff for hip dysplasia [1, 10]. However, details related to the specific anatomical characteristics of the included patients were not investigated in the present study. Since other anatomical variables, such as femoral torsion and tendon insertional anatomy were observed to influence outcomes after iliopsoas tenotomy, future studies incorporating radiographic and anatomical assessments would be valuable to better delineate their role.

Other limitations of this study exist: the retrospective nature of this study lowers its level of evidence and does not permit analysis of the progressive evolution of clinical outcomes over time. Plus, the small cohort of included patients leads to a low statistical power and individual data points could have a disproportionate impact on the results: a single outlier or unique data point might either obscure or falsely suggest a trend that wouldn't exist in a larger group. The absence of a control group further limits the strength of causal inferences. Furthermore, the inclusion of patients with concomitant intra‐articular procedures introduces a bias and complicates the attribution of clinical improvement solely to the tenotomy. Then, the absence of Beighton Index assessment represents a limitation, as it precluded evaluation of generalised ligamentous laxity as a potential factor in hip microinstability.

Several strengths of this study should be highlighted. The long‐term follow‐up period provides insight into the durability of arthroscopic iliopsoas release. Moreover, all procedures were performed in a single institution by one experienced surgeon, ensuring consistency in surgical technique and rehabilitation.

In conclusion, transcapsular endoscopic iliopsoas tenotomy appears to be an effective surgical option for patients with symptomatic snapping hip and iliopsoas impingement resistant to conservative treatment. The procedure demonstrates excellent long‐term outcomes and high patient satisfaction. Future prospective studies with larger cohorts, anatomical variables assessment and comparative designs are needed to further refine patient selection criteria and optimise surgical outcomes.

## AUTHOR CONTRIBUTIONS

*Conce ptualisation and design of the study*: Mattia Loppini, Federico Della Rocca and Guido Grappiolo. *Methodology*: Mattia Loppini and Marco Minelli. *Validation*: Marco Minelli. *Formal analysis*: Marco Minelli and Katia Chiappetta. *Investigation*: Mattia Loppini, Marco Minelli and Federico Della Rocca. *Data curation*: Marco Minelli, Francesco La Camera and Katia Chiappetta. *Writing – original draft preparation*: Marco Minelli. *Writing – review and editing*: Mattia Loppini and Francesco La Camera. *Supervision*: Guido Grappiolo. All authors have read and agreed to the published version of the manuscript.

## CONFLICT OF INTEREST STATEMENT

The authors declare no conflict of interest.

## ETHICS STATEMENT

Institutional Board Committee (protocol number 618/17 IRCCS Istituto Clinico Humanitas).

## Data Availability

None declared.

## References

[1] Atzmon R , Safran MR . Arthroscopic Treatment of mild/borderline hip dysplasia with concomitant femoroacetabular impingement: literature review. Curr Rev Musculoskelet Med. 2022;15(4):300–310. 10.1007/s12178-022-09765-4 PMC9276885 35708882

[2] Audenaert EA , Khanduja V , Claes P , Malviya A , Steenackers G . Mechanics of psoas tendon snapping. a virtual population study. Front Bioeng Biotechnol. 2020;8:264. 10.3389/fbioe.2020.00264 PMC7118580 32292780

[3] Benes M , Bartak V , Uhlik J , Novotny T , Rybakova A , Kachlik D , et al. Surgical anatomy of the anterior musculocapsular complex of the hip: a macroscopic and microscopic anatomical reappraisal. Anat Cell Biol. 2025;58(2):155–165. 10.5115/acb.24.329 PMC12178697 40194840

[4] Brandenburg JB , Kapron AL , Wylie JD , Wilkinson BG , Maak TG , Gonzalez CD , et al. The functional and structural outcomes of arthroscopic iliopsoas release. Am J Sports Med. 2016;44(5):1286–1291. 10.1177/0363546515626173 26872894

[5] Chandrasekaran S , Close MR , Walsh JP , Chaharbakhshi EO , Lodhia P , Mohr MR , et al. Arthroscopic technique for iliopsoas fractional lengthening for symptomatic internal snapping of the hip, iliopsoas impingement lesion, or both. Arthrosc Tech. 2018;7(9):e915–e919. 10.1016/j.eats.2018.06.001 PMC6153307 30258772

[6] Contreras MEK , Dani WS , Endges WK , De Araujo LCT , Berral FJ . Arthroscopic treatment of the snapping iliopsoas tendon through the central compartment of the hip: a pilot study. J Bone Joint Surg Br. 2010;92(6):777–780. 10.1302/0301-620X.92B6.22797 20513872

[7] Coulomb R , Nougarede B , Maury E , Marchand P , Mares O , Kouyoumdjian P . Arthroscopic iliopsoas tenotomies: a systematic review of surgical technique and outcomes. HIP Int. 2022;32(1):4–11. 10.1177/1120700020970519 33226846

[8] Dettoni F , Pellegrino P , La Russa MR , Bonasia DE , Blonna D , Bruzzone M , et al. Validation and cross cultural adaptation of the Italian version of the Harris Hip Score. HIP Int. 2015;25(1):91–97. 10.5301/hipint.5000184 25198299

[9] Domb BG , Shindle MK , McArthur B , Voos JE , Magennis EM , Kelly BT . Iliopsoas impingement: a newly identified cause of labral pathology in the hip. HSS J. 2011;7(2):145–150. 10.1007/s11420-011-9198-z PMC3145856 22754415

[10] Domb BG , Wallace IA , Becker N . Editorial commentary: arthroscopic treatment of mild hip dysplasia can result in excellent outcome and avoid more invasive periacetabular osteotomy. Arthrosc ‐ J Arthrosc Relat Surg. 2025;41(2):226–228. 10.1016/j.arthro.2024.10.023 39481668

[11] Engelken F , Wassilew GI , Köhlitz T , Brockhaus S , Hamm B , Perka C , et al. Assessment of fatty degeneration of the gluteal muscles in patients with THA using MRI: reliability and accuracy of the Goutallier and quartile classification systems. J Arthroplasty. 2014;29(1):149–153. 10.1016/j.arth.2013.04.045 23743509

[12] Fabricant PD , Bedi A , De La Torre K , Kelly BT . Clinical outcomes after arthroscopic psoas lengthening: the effect of femoral version. Arthrosc ‐ J Arthrosc Relat Surg. 2012;28(7):965–971. 10.1016/j.arthro.2011.11.028 22305298

[13] Fischer CS , Kühn JP , Ittermann T , Schmidt CO , Gümbel D , Kasch R , et al. What are the reference values and associated factors for center‐edge angle and alpha angle? A population‐based study. Clinical Orthopaedics & Related Research. 2018;476(11):2249–2259. 10.1097/CORR.0000000000000410 PMC6259987 30024461

[14] Gouveia K , Shah A , Kay J , Memon M , Simunovic N , Cakic JN , et al. Iliopsoas tenotomy during hip arthroscopy: a systematic review of postoperative outcomes. Am J Sports Med. 2021;49:817–829. 10.1177/0363546520922551 32628861

[15] Hain KS , Blankenbaker DG , De Smet AA , Keene JS , Del Rio AM . MR appearance and clinical significance of changes in the hip muscles and iliopsoas tendon after arthroscopic iliopsoas tenotomy in symptomatic patients. HSS J. 2013;9(3):236–241. 10.1007/s11420-013-9361-9 PMC3772171 24426875

[16] Harris JD . Editorial commentary: caveat flexor‐to release or not to release the iliopsoas, that is the question. Arthrosc ‐ J Arthrosc Relat Surg. 2018;34(6):1851–1855. 10.1016/j.arthro.2018.04.010 29804606

[17] Hoskins JS , Burd TA , Allen WC . Surgical correction of internal coxa saltans: a 20‐year consecutive study. Am J Sports Med. 2004;32(4):998–1001. 10.1177/0363546503260066 15150049

[18] Hwang A , Martinez M , Cora Jones CM , Giordano B . Multifid iliopsoas tendons are more common in patients with painful snapping iliopsoas tendons. Arthrosc Sports Med Rehabil. 2023;5(5):100780. 10.1016/j.asmr.2023.100780 PMC10400859 37546385

[19] Ilizaliturri VM , Buganza‐Tepole M , Olivos‐Meza A , Acuna M , Acosta‐Rodriguez E . Central compartment release versus lesser trochanter release of the iliopsoas tendon for the treatment of internal snapping hip: a comparative study. Arthrosc ‐ J Arthrosc Relat Surg. 2014;30(7):790–795. 10.1016/j.arthro.2014.03.008 24793208

[20] Ilizaliturri VM , Byrd JWT , Sampson TG , Guanche CA , Philippon MJ , Kelly BT , et al. A geographic zone method to describe intra‐articular pathology in hip arthroscopy: cadaveric study and preliminary report. Arthrosc ‐ J Arthrosc Relat Surg. 2008;24(5):534–539. 10.1016/j.arthro.2007.11.019 18442685

[21] Kuhns BD , Wallace IA , Kahana‐Rojkind AH , Domb BG . Tendon‐sparing iliopsoas tunnel deepening with anterior labral refixation for the painful snapping hip. Arthrosc Tech. 2025;14(5):103440. 10.1016/j.eats.2025.103440 PMC12177478 40548045

[22] Lin B , Bartlett J , Lloyd TD , Challoumas D , Brassett C , Khanduja V . Multiple iliopsoas tendons: a cadaveric study and treatment implications for internal snapping hip syndrome. Arch Orthop Trauma Surg. 2022;142(6):1147–1154. 10.1007/s00402-021-04009-5 PMC9110434 34347120

[23] Longstaffe R , Hendrikx S , Naudie D , Willits K , Degen RM . Iliopsoas release: a systematic review of clinical efficacy and associated complications. Clin J Sport Med. 2021;31(6):522–529. 10.1097/JSM.0000000000000784 32032164

[24] Maldonado DR , Krych AJ , Levy BA , Hartigan DE , Laseter JR , Domb BG . Does iliopsoas lengthening adversely affect clinical outcomes after hip arthroscopy? A multicenter comparative study. Am J Sports Med. 2018;46(11):2624–2631. 10.1177/0363546518785966 30074842

[25] Mayne AIW , Al‐Shahwani A , Gosling L , Wall P , Politis A , McBryde C . Arthroscopic iliopsoas release following hip arthroplasty surgery: a successful procedure but beware of instability! HIP Int. 2025;35(4):370–376. 10.1177/11207000251339063 40384093

[26] Mimura T , Furuya Y , Kumagai K , Amano Y , Miyahara S , Uemura R , et al. The ability of plain radiography to accurately describe the bone surface at the head‐neck junction of the femur: a study using human bone models. J Hip Preserv Surg. 2024;12(1):65–73. 10.1093/jhps/hnae048 PMC12051853 40331075

[27] Nelson IR , Keene JS . Results of labral‐level arthroscopic iliopsoas tenotomies for the treatment of labral impingement. Arthrosc ‐ J Arthrosc Relat Surg. 2014;Jun 30(6):688–694. 10.1016/j.arthro.2014.02.027 24704071

[28] Sugrañes J , Jackson GR , Warrier AA , Allahabadi S , Chahla J . Snapping hip syndrome: pathoanatomy, diagnosis, nonoperative therapy, and current concepts in operative management. JBJS Rev. 2023;11(6). 10.2106/JBJS.RVW.23.00005 37289915

[29] Torre M , Luzi I , Mirabella F , Del Manso M , Zanoli G , Tucci G , et al. Cross‐cultural adaptation and validation of the Italian version of the Hip Disability and Osteoarthritis Outcome Score (HOOS). Health Qual Life Outcomes. 2018;16(1):115. 10.1186/s12955-018-0935-6 PMC5987663 29866107

[30] Tsukada S , Niga S , Nihei T , Imamura S , Saito M , Hatanaka J . Iliopsoas disorder in athletes with groin pain: prevalence in 638 consecutive patients assessed with MRI and clinical results in 134 patients with signal intensity changes in the iliopsoas. JB JS Open Access. 2018;3(1):e0049. 10.2106/JBJS.OA.17.00049 PMC6132908 30229237

[31] Walker P , Ellis E , Scofield J , Kongchum T , Sherman WF , Kaye AD . Snapping hip syndrome: a comprehensive update. Orthop Rev. 2021;13(2):25088. 10.52965/001c.25088 PMC8567760 34745476

[32] Walczak BE , Blankenbaker DG , Tuite MR , Keene JS . Magnetic resonance imaging appearance of the hip musculature after arthroscopic labral‐level iliopsoas tenotomies. Orthop J Sports Med. 2017;5(5):2325967117707498. 10.1177/2325967117707498 PMC5448789 28596974

